# Intimate partner violence against women in Ethiopia: An umbrella review of systematic reviews and meta-analyses

**DOI:** 10.1371/journal.pone.0362748

**Published:** 2026-11-11

**Authors:** Teklehaimanot Gereziher Haile, Negasi Asres, Dawit Gebregziabher, Birhane Fissahaye, Yemane Leake, Girmay Teklay, Haftu Berhe Gebru

**Affiliations:** 1 Department of Maternity and Neonatal Nursing, School of Nursing, College of Health Sciences and Comprehensive Specialized Hospital, Aksum University, Aksum, Tigray, Ethiopia; 2 Department of Epidemiology and Biostatistics, School of Public Health, College of Health Sciences and Comprehensive Specialized Hospital, Aksum University, Aksum, Tigray, Ethiopia; 3 School of Nursing, College of Health Sciences and Comprehensive Specialized Hospital, Aksum University, Aksum, Tigray, Ethiopia; 4 School of Medicine, College of Health Sciences and Comprehensive Specialized Hospital, Aksum University, Aksum, Tigray, Ethiopia; 5 Department of Pediatric and Child Health Nursing, School of Nursing, College of Health Sciences and Comprehensive Specialized Hospital, Aksum University, Aksum, Tigray, Ethiopia; 6 School of Nursing, College of Health Science, Mekelle University, Mekelle, Tigray, Ethiopia; Curtin University Bentley Campus: Curtin University, AUSTRALIA

## Abstract

**Introduction:**

Intimate partner violence is a major global public health concern and a serious human rights violation, involving physical, sexual, and psychological abuse within intimate relationships. Worldwide, about one in three women experiences intimate partner violence during their lifetime. Accordingly, this study aims to estimate the overall prevalence and identify the key predictors of intimate partner violence in Ethiopia.

**Methods:**

A comprehensive systematic search was carried out in PubMed, Web of Science, Google Scholar, and African Journals Online to identify relevant systematic reviews and meta-analyses on the prevalence and predictors of intimate partner violence against women in Ethiopia. The methodological quality of the included studies was assessed using the AMSTAR-2. A random-effects meta-analysis was conducted using STATA version 18 to generate pooled estimates.

**Results:**

This umbrella review synthesized evidence from six systematic reviews and meta-analyses comprising 80 primary studies involving 43,785 women. The overall prevalence of intimate partner violence was 35% (95% CI: 28–42%), with substantial heterogeneity across reviews (I^2^ = 83.48%). It also revealed a prevalence of 32% (95% CI: 26–37%) among pregnant women and 33% (95% CI: 25–40%) during the COVID-19 pandemic. The prevalence of specific forms of violence was estimated at 18% (95% CI: 14–22%) for sexual violence, 19% (95% CI: 15–23%) for physical violence, and 27% (95% CI: 23–31%) for psychological violence. Key predictors of IPV included both partners’ and women’s educational status, partner alcohol consumption, women’s decision-making power, substance use, and income loss during the COVID-19 pandemic, with all associations found to be statistically significant.

**Conclusion:**

This umbrella review shows that intimate partner violence remains a major public health issue in Ethiopia, affecting over one-third of women. Strengthening policy implementation and expanding survivor-centered services are essential to reduce IPV and advance Sustainable Development Goal 5.

## Introduction

Intimate partner violence (IPV) is a major global public health concern and a serious human rights violation, encompassing physical, sexual, and psychological abuse within intimate relationships [1]. Survivors experience significantly poorer physical and mental health outcomes [2,3]. Although violence against women occurs in various settings, including workplaces, schools, and communities, intimate partner violence (IPV) remains the most common form [4,5].

IPV is a global problem affecting women in all countries, although its prevalence varies considerably across regions [6,7]. Globally, approximately one in three women experiences at least one form of IPV during her lifetime [1]. However, the burden is unevenly distributed, with the World Health Organization (WHO) reporting prevalence estimates of 23% in high-income countries, compared with 37% in the Eastern Mediterranean, 38% in South-East Asia, and 36.3% in sub-Saharan Africa [8]. Ethiopia reflects the high burden of IPV observed in many African countries, with previous studies reporting lifetime prevalence estimates ranging from 20% to 78%, depending on the region [9].

The consequences of IPV are devastating, resulting in severe short- and long-term physical injuries, mental health problems, including depression, anxiety, and post-traumatic stress disorder (PTSD), and an increased risk of suicide [8]. Beyond its direct health effects, IPV has substantial social and economic consequences for women, their families, and society. The trauma associated with abuse often leads to social isolation, absenteeism from work, reduced engagement in daily activities, and impaired ability to care for oneself and one's family [10,11].

According to recent data from the Ethiopian Demographic and Health Survey (DHS), IPV remains widespread in Ethiopia. The report indicates that 27% of ever-married women of reproductive age experienced IPV in the year preceding the survey, while 4% experienced violence during pregnancy. A critically concerning finding is that over two-thirds of IPV survivors never disclose the abuse or seek help [12,13]. Beyond physical and sexual violence, coercive control is also rampant; data from the Central Statistical Agency (CSA) and ICF (2017) show that a majority (57%) of women have been subjected to at least one form of marital controlling behavior by a partner [13].

Preventing violence against women is fundamental to achieving gender equality and women's empowerment, as recognized in Sustainable Development Goal 5 (SDG-5), adopted by the United Nations (UN), which aims to eliminate all forms of violence against women and girls [14]. In response, the WHO and the UN developed the RESPECT Women framework to guide evidence-based, multisectoral strategies for preventing violence against women [11,15]. Consistent with these global initiatives, the Ethiopian government, in collaboration with UN Women and the United Nations Children's Fund (UNICEF), launched a five-year National Action Plan (2021/22–2025/26) to prevent and respond to violence against women and children. The plan aims to strengthen reporting systems, expand survivor-centered support services, and protect the rights of women and children to live free from violence [16].

The WHO ecological framework provides a widely accepted conceptual model for understanding IPV by recognizing that violence results from the interaction of factors operating at the individual, relationship, community, and societal levels. Individual-level factors include educational attainment, substance use, and prior exposure to violence, whereas relationship-level factors include partner alcohol consumption, controlling behaviors, and women's decision-making power. Community- and societal-level influences, such as social norms, gender inequality, poverty, and weak institutional support, further shape women's vulnerability to IPV [17–19]. This ecological perspective highlights the multifactorial nature of IPV and emphasizes that effective prevention requires coordinated interventions across multiple levels rather than focusing solely on individual behaviors.

Although developing countries, including Ethiopia, have adopted legal frameworks to protect women's rights, substantial implementation gaps remain, hindering efforts to prevent violence against women [20]. Several systematic reviews and meta-analyses have examined the prevalence and predictors of IPV in Ethiopia; however, their findings vary because of differences in study populations, settings, outcome definitions, and methodological approaches. To the best of our knowledge, no umbrella review has comprehensively synthesized this evidence. By integrating findings from existing systematic reviews and meta-analyses, an umbrella review provides a higher level of evidence to comprehensively assess the burden of IPV and the consistency of its reported predictors.

The findings can inform policy development, healthcare planning, clinical practice, and the design of evidence-based strategies to prevent and respond to IPV. Therefore, this umbrella review aimed to estimate the overall prevalence of IPV, synthesize evidence on the prevalence of its major forms and among key population subgroups, and identify the major predictors of IPV among women in Ethiopia.

## Materials and methods

This umbrella review was undertaken to comprehensively synthesize evidence from published systematic reviews and meta-analyses on IPV against women in Ethiopia, providing a robust evidence base to inform clinical practice, public health interventions, and policy development [21]. The review was conducted in accordance with established methodological guidance for umbrella reviews [22] and reported following the Preferred Reporting Items for Systematic Reviews and Meta-Analyses (PRISMA) guidelines [23] (S1 Table). The review protocol was registered with PROSPERO on 29 July 2026 (Registration No. CRD420261463983). As the registration occurred after the review activities had commenced, including screening, data extraction, and analysis, the registration was retrospective.

### Eligibility criteria

This umbrella review included all eligible SRMAs of observational studies (cross-sectional, cohort, or case-control) that reported the prevalence and/or predictors of IPV among women in Ethiopia. Eligibility criteria were defined according to the population, outcome, study design, language, and publication status. Specifically, eligible studies included women in Ethiopia, reported the prevalence and/or predictors of IPV, were systematic reviews and meta-analyses of observational studies, were published in English, and had full-text articles available. No restrictions were placed on publication date up to the search cutoff (10 February 2026). Both published and unpublished SRMAs were eligible for inclusion.

We excluded narrative reviews, scoping reviews, editorials, letters, conference abstracts, methodological papers, and systematic reviews without meta-analysis. Reviews with poorly described methodologies, including those lacking a clearly defined research question, search strategy, or study selection process, were also excluded. In addition, studies that did not report the prevalence or predictors of IPV among women in Ethiopia were excluded.

### Assessment of overlap of primary studies

To evaluate overlap of primary studies across the included systematic reviews and meta-analyses, a citation matrix was constructed and the Corrected Covered Area (CCA) was calculated. The CCA was interpreted as slight (0–5%), moderate (6–10%), high (11–15%), or very high (>15%) overlap, as recommended in the umbrella review methodology literature [24].

### Search strategy

A comprehensive literature search was independently conducted by two authors (TGH and NA) to identify published and unpublished systematic reviews and meta-analyses on IPV against women in Ethiopia. Electronic databases, including PubMed/MEDLINE, Web of Science, Google Scholar, and African Journals Online (AJOL), were systematically searched from inception to 10 February 2026.

The search strategy combined Medical Subject Headings (MeSH) and free-text terms related to IPV, women, Ethiopia, systematic reviews, meta-analyses, and associated predictors using the Boolean operators AND and OR. Database-specific search strategies were adapted according to the indexing system and search functionality of each database.

To identify unpublished studies (grey literature), we conducted a systematic manual search of Ethiopian university institutional repositories using Google and repository-specific search functions. Where applicable, the same keywords and Boolean operators used for the electronic database searches were applied. In addition, the reference lists of all eligible reviews were manually screened to identify further relevant studies. The complete search strategies for all databases are provided in (Table 1).

**Table 1:** Search strategies used for each electronic database.

Database	Search strategy
PubMed/MEDLINE	((“Intimate Partner Violence”[Mesh] OR “intimate partner violence” OR IPV OR “domestic violence” OR “spousal violence”) AND (women OR woman OR female) AND (Ethiopia) AND (“systematic review” OR “meta-analysis”) AND (predictor* OR determinant* OR “associated factor*”))
Web of Science	TS=(“intimate partner violence” OR IPV OR “domestic violence” OR “spousal violence”) AND TS=(women OR woman OR female) AND TS=(Ethiopia) AND TS=(“systematic review” OR “meta-analysis”) AND TS=(predictor* OR determinant* OR “associated factor*”)
Google Scholar	“intimate partner violence” Ethiopia systematic review meta-analysis women predictors
African Journals Online (AJOL)	“intimate partner violence” AND Ethiopia AND women AND (“systematic review” OR “meta-analysis”)
Manual search	Reference lists of all eligible studies and relevant review articles were screened. Grey literature was identified through Google searches of Ethiopian university institutional repositories.

The PubMed/MEDLINE search strategy was as follows:

((“Intimate Partner Violence”[Mesh] OR “intimate partner violence” OR IPV OR “domestic violence” OR “spousal violence”) AND (women OR woman OR female) AND (Ethiopia) AND (“systematic review” OR “meta-analysis”) AND (predictor* OR determinant* OR “associated factor*”)).

### Selection process

Following the database searches, all identified records were exported into EndNote 21 to eliminate duplicate publications. Two independent reviewers (DG and BF) then screened the remaining articles in a two-stage process: first by title and abstract, and subsequently by a full-text review against the pre-defined inclusion criteria. To ensure consistency, any discrepancies between the two reviewers regarding the eligibility of studies were resolved through discussion and consensus with a third reviewer (HBG).

### Data extraction

To ensure rigorous quality assessment and synthesis, data were extracted in duplicate from all included SRMAs. Using a standardized form in Microsoft Excel 2024, the two reviewers (TGH and GT) collected key information from each SRMA. This included bibliographic details (first author and publication year), methodological characteristics (search strategy, number and design of primary studies, sample size, risk of bias tool), and outcome data (reported prevalence of IPV, key predictors, and significant findings).

### Outcomes

The primary outcome of this umbrella review was the overall prevalence of IPV among women in Ethiopia. The secondary outcome was the association between IPV and its predictors. In addition, the review was planned a priori to synthesize evidence on the prevalence of the major forms of IPV (psychological, physical, and sexual violence) and among key population subgroups, including pregnant women and women during the COVID-19 pandemic, because these represent important public health populations and outcomes that were consistently reported across the included systematic reviews.

### Quality assessment of the systematic review and meta‑analyzed studies

The methodological quality of all included systematic reviews and meta-analyses (SRMAs) was assessed using the AMSTAR-2 (A Measurement Tool to Assess Systematic Reviews) instrument [25]. This tool comprises 16 domains, 7 of which are critical. These critical domains evaluate protocol registration, literature search adequacy, justification for study exclusions, risk of bias in included studies, appropriateness of meta-analytical methods, consideration of risk of bias in interpretation, and assessment of publication bias.

Two independent authors (TGH and YL) scored each included SRMA on all 16 items using the ratings: “Yes,” “Partial Yes,” “No,” or “No meta-analysis conducted.” Disagreements were resolved by consulting a third author (NA). Following AMSTAR-2 guidelines, each SRMA was then assigned an overall quality rating: High (no or one non-critical weakness), Moderate (no critical flaws but multiple non-critical flaws), Low (one critical flaw), or Critically Low (more than one critical flaw).

### Data analysis

The data were analyzed using STATA version 18. The findings were synthesized through a narrative summary followed by an umbrella meta-analysis. The overall pooled prevalence of IPV and the associations between identified predictors and IPV were estimated using random-effects models (DerSimonian–Laird for prevalence estimates and Hedges’ method for effect sizes), with the results presented as forest plots. Statistical heterogeneity was assessed using the I^2^ statistic, with values ≥75% considered indicative of substantial heterogeneity [26].

Before quantitative synthesis, overlap of primary studies across the included systematic reviews and meta-analyses was evaluated using a citation matrix and the CCA. This assessment was undertaken to quantify the extent of overlap and to inform the interpretation of the pooled estimates in light of the potential for double counting of primary studies. Owing to the inclusion of fewer than ten systematic reviews and meta-analyses, publication bias was not formally assessed, as statistical methods for detecting publication bias have limited reliability with fewer than ten studies.

### Ethical approval and consent to participate

Not applicable.

## Results

### Searching finding

The initial literature search yielded 419 records. After removing 115 duplicates, 304 articles underwent title and abstract screening, resulting in the exclusion of 278 irrelevant studies. A full-text review of the remaining 26 articles was conducted for eligibility. Of these, 20 were excluded as they did not meet the outcome criteria, primarily because they were not systematic reviews or meta-analyses focused on the specific topic of IPV in Ethiopia. Therefore, this umbrella review included a final total of 6 systematic reviews and meta-analyses for data synthesis and analysis (Fig 1).

**Fig 1:**
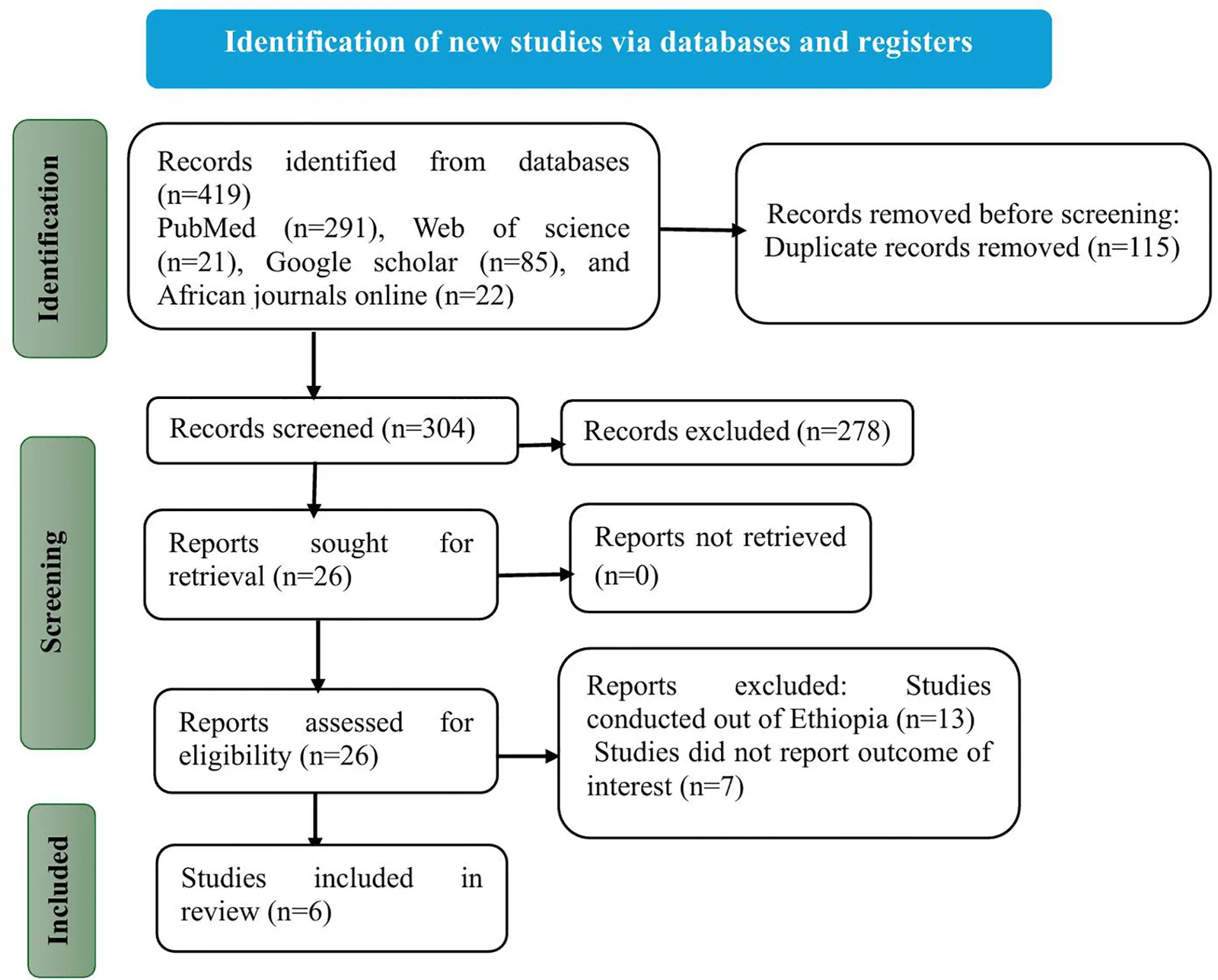
PRISMA 2020 flow diagram for searching, screening and identification of SRMA studies.

### Characteristics of included review studies

This umbrella reviews synthesized evidence from six systematic reviews and meta-analyses (SRMAs) derived from observational studies. Collectively, the included SRMAs comprised 80 primary studies (75 cross-sectional and 5 case-control) involving 43,785 women, of whom 15,147 had experienced IPV. The median sample size across the SRMAs was 6,839 participants, with a median of 2,232 IPV cases. Five of the six SRMAs were published within the last five years. While one review focused exclusively on the prevalence of IPV, the remaining five examined both its prevalence and associated predictors. The reported pooled prevalence of IPV across the reviews ranged from 26.1% (95% CI: 20.0–32.3) to 51.5% (95% CI: 44.1–58.9) (Table 2).

**Table 2:** Characteristics of the included systematic reviews and meta-analyses on intimate partner violence against women in Ethiopia.

Authors (Year)	Publication period of primary studies	Included Studies	Design	Search strategies	Sample size	Reported Prevalence	Risk of bias	Reported factors	Quality assessment
Bifftu BB, Guracho YD [27],	2003-2020	26	26 Cross-Sectional	PubMed/Medline, CINAHL, Scopus, HINARI (research4life), AJOL, IRIS, and AIM, national and international institutions and research centers, and web of science (Google and Google scholar)	13, 912	36.75% (29.90% −43.60%) I2 = 96.5%	The quality of the included studies was clearly appraised	participants who have lack of formal education (AOR = 3.88; 95% CI: 1.48, 6.27), childhood violence (AOR = 3.14; 95% CI: 1.37, 4.90), rural residency (AOR = 2.48; 95% CI: 1.29, 3.67), low decision-making power (AOR = 1.30; 95% CI: 1.03, 1.58), family history of violence (AOR = 1.68; 95% CI: 1.14, 2.22), attitude toward domestic violence (AOR = 12.92; 95% CI: 6.58, 19.25), unplanned pregnancy (AOR = 1.77; 95% CI: 1.48, 2.05), late initiation of antenatal care (AOR = 1.30; 95% CI: 1.15, 1.44), partner alcohol use (AOR = 2.19; 95% CI: 1.66, 2.71), partner’s unwanted pregnancy (AOR = 1.20; 95% CI: 1.03, 1.36), Unwanted pregnancy by women (AOR = 1.54; 95% CI: 1.00, 2.08), partner khat use (AOR = 1.73; 95% CI: 1.42, 2.05), and No partner education (AOR = 1.10; 95% CI: 0.93, 1.28)	NOS
Belay AS, et al.[28],	2020-2023	12	12 Cross-Sectional	PubMed/Medline, Cochrane Library, Virtual Health Library Regional Portal, Health Inter-Network Access to Research Initiative (HINARI) or (research4life), Google Scholar, and grey literatures were checked using national and international institutional repositories	7,397	33.53% (95% CI 22.96% to 44.1%) I2 = 99.2%	The quality of the included studies was clearly appraised	Young women’s age (AOR = 3.24; 95%CI: 0.6, 5.8), Lack of formal education (AOR = 2.63; 95%CI: 1.7, 3.6), Partner’s substance use (AOR = 2.07; 95%CI: 1.5, 2.7), Partner’s alcohol use (AOR = 2.82; 95%CI: 1.1, 4.6), Partner not attending formal education (AOR = 3.38; 95%CI: 2.2, 4.6), and Women’s loss of income due to COVID-19 (AOR = 9.69; 95%CI: 4.9, 14.5)	JBI
Alebel et al.[29],	2013-2017	8	7 Cross-Sectional and 1 Case control	PubMed/MEDLINE, EMBASE, CINAHL, Google Scholar, Science Direct and Cochrane Library	2,756	26.1% (95%CI: 20, 32.3) I2 = 93.0	The quality of the included studies was clearly appraised	Mother’s educational status (OR: 2.1; 95% CI: 1.1, 3.7), intimate partners’ educational status (OR: 3.5; 95%CI: 1.4, 8.5), and intimate partners’ alcohol use (OR: 11.4; 95%CI: 2.3, 56.6)	NOS
Alemu et al.[30],	2020-2022	10	10 Cross-Sectional	PubMed, Google, Google Scholar, Cochrane Library, CINAHL, and searched the reference lists of the included studies	6,280	31.60% (95% CI: 21.10, 42.11) I2 = 99.1%	The quality of the included studies was clearly appraised	Partner alcohol use (AOR = 1.93; 95% CI: 1.60, 2.33), income lost during the COVID-19 pandemic (AOR = 9.98; 95% CI: 6.35, 15.70), partners who had no formal education (AOR = 2.03; 95% CI: 1.57, 2.63), and decisions made by their partner only (AOR = 1.82; 95% CI: 1.33, 2.50)	JBI
Belay et al.[31],	2014-2022	17	14 Cross-Sectional and 3 Case control	PubMed, Google Scholar, Scopus, Embase, CINAHL, and websites of universities and research institutes	10,736	32.23% (95% CI: 28.02, 36.45) I2 = 98.4%	The quality of the included studies was clearly appraised	NA	NOS
Alemu SS. et al.[32],	2016-2023	7	6 Cross-Sectional and 1 Case control	MEDLINE/PubMed, Google Scholar, EMBASE, HINARI, Cochrane Library, ProQuest, African Journals Online, and online university repositories, and reference lists of previous studies	2,704	51.45% (95% CI: 44.05, 58.85) I2 = 93.33%	The quality of the included studies was clearly appraised	Having multiple partners (OR = 2.12; 95% CI: 1.40, 2.84), women experiencing controlling behavior by a partner (OR = 5.95; 95% CI: 3.56, 8.35), partners who drink alcohol (OR = 2.23; 95% CI: 1.45, 3.01), and partners involved in a physical fight with another man (OR = 2.02; 95% CI: 1.10, 2.94)	JBI

*N/A: Not Applicable, NOS: Newcastle-Ottawa Scale, JBI: Joanna Briggs Institute.*

To assess the degree of overlap among the included SRMAs, we developed a citation matrix of the primary studies included in each review (S2 Table). The matrix identified 50 unique primary studies, involving a total of 26,220 participants, which contributed to 80 study occurrences across the six included systematic reviews and meta-analyses. This indicates that several primary studies were included in more than one review. The CCA was 12%, indicating a high degree of overlap among the included reviews. The overlap was taken into account when interpreting the pooled estimates, and the potential for partial double counting of evidence was acknowledged as a limitation. Therefore, the pooled findings should be interpreted with appropriate caution because overlapping primary studies may have contributed disproportionately to some estimates.

### Quality assessment

The methodological quality of the included systematic reviews and meta-analyses was assessed using the AMSTAR-2 critical appraisal tool [25]. Overall confidence ratings were assigned according to the AMSTAR-2 guidance based on the presence of critical and non-critical weaknesses. The assessment identified several methodological strengths, including clearly defined review questions, comprehensive literature searches, duplicate study selection and data extraction, and appropriate statistical methods for meta-analysis.

However, several methodological shortcomings were also identified. The most common critical flaws were the absence of prospective protocol registration and failure to provide a list of excluded studies with justification. Common non-critical flaws included incomplete use of the PICO framework to define the eligibility criteria. Detailed AMSTAR-2 ratings, including the overall confidence rating for each review, are presented in (S3 Table).

These findings indicate that the included reviews provide generally reliable evidence; however, the identified methodological limitations should be considered when interpreting the synthesized findings, as they may reduce confidence in some estimates.

### Meta-analysis of the prevalence of intimate partner violence

The analysis of six meta-analyses showed that the overall prevalence of IPV against women in Ethiopia was 35% (95% CI: 28–42%) (Fig 2). The heterogeneity assessment revealed a high level of variability across studies, with an I² of 83.48% (p = 0.00). To address this substantial heterogeneity, a random-effects model was applied, which considers both within-study and between-study differences, producing a more conservative and widely generalizable pooled estimate.

**Fig 2:**
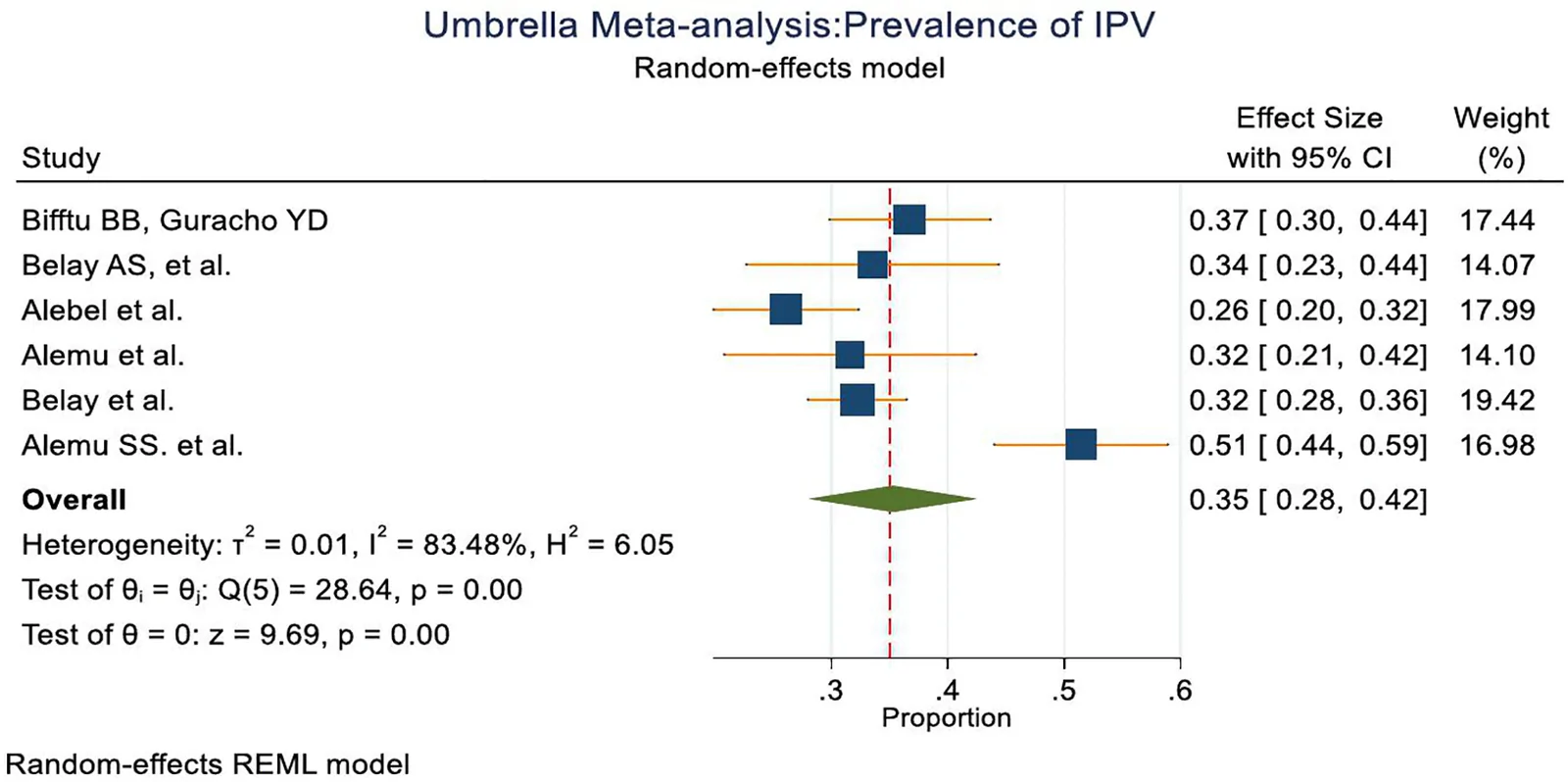
Umbrella review on the prevalence of intimate partner violence against women in Ethiopia.

The substantial heterogeneity observed across the included reviews likely reflects differences in study populations, geographic regions, outcome definitions, measurement instruments, and study periods, including the inclusion of studies conducted before and during the COVID-19 pandemic. Ethiopia's considerable sociocultural and regional diversity may further influence both the occurrence and reporting of IPV.

Based on three meta-analyses, the pooled prevalence of IPV among pregnant women in Ethiopia was 32% (95% CI: 26–37%) (Fig 3). The heterogeneity analysis yielded an I^2^ of 65.05% (p = 0.07), indicating moderate variability across the studies, with much of the observed differences likely attributable to sampling error rather than true variation between studies.

**Fig 3:**
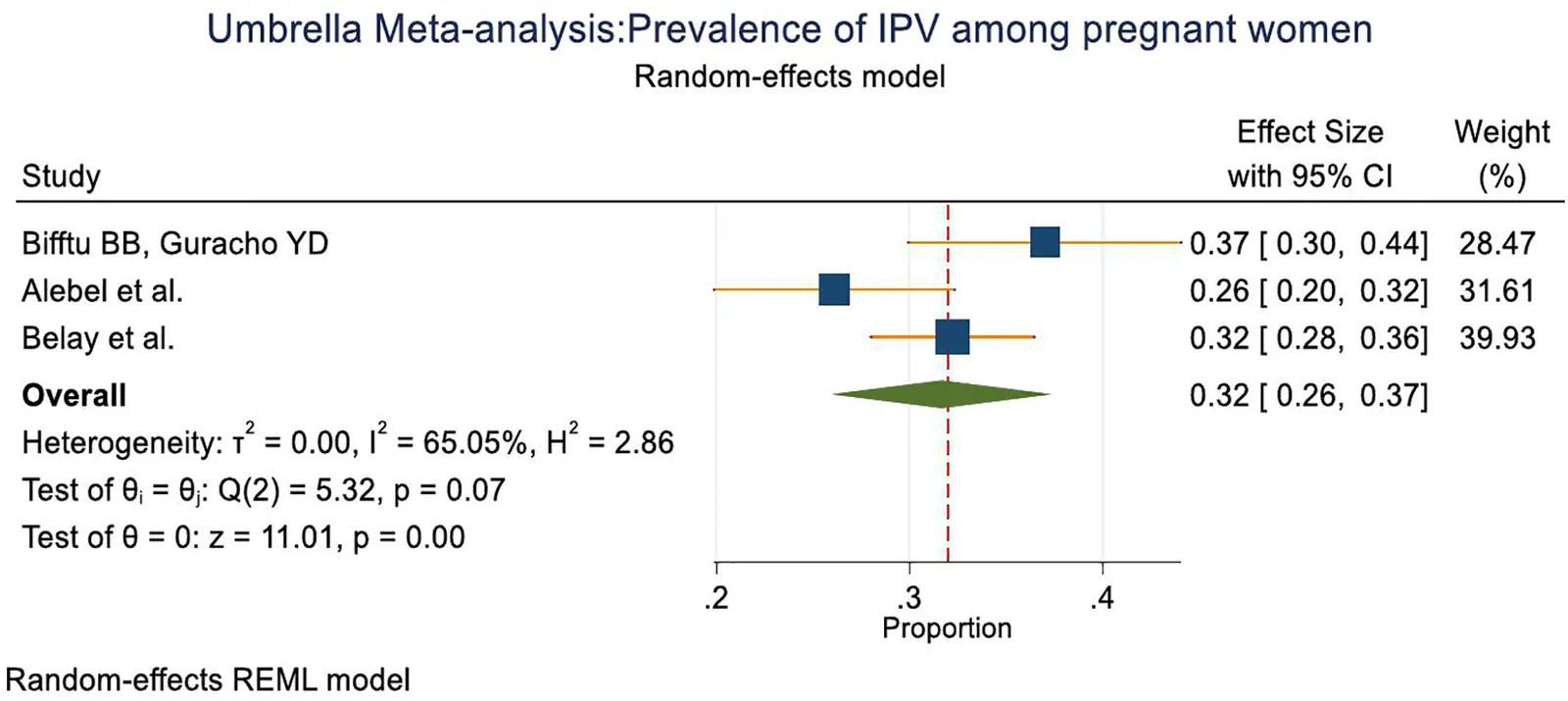
Pooled prevalence of intimate partner violence among pregnant women across included meta-analyses.

During the COVID-19 pandemic, the pooled prevalence of IPV in Ethiopia was estimated at 33% (95% CI: 25–40%) (Fig 4). The heterogeneity analysis showed an I^2^ value of 0.00% (p = 0.00), indicating no observed heterogeneity among the included studies.

**Fig 4:**
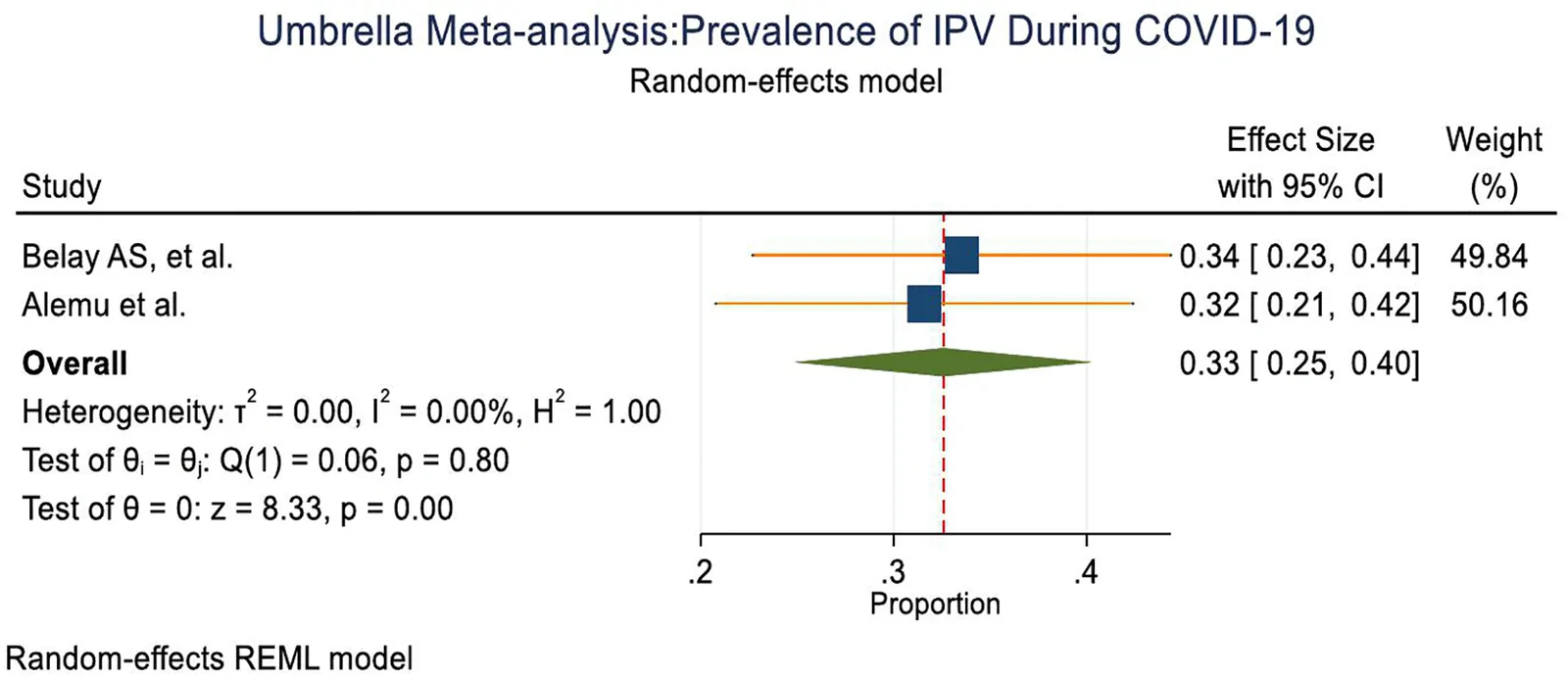
Pooled prevalence of intimate partner violence against women during the COVID-19 pandemic across included meta-analyses.

Four included studies showed a pooled prevalence of partner sexual violence was 18% (95% CI: 14–22%) (Fig 5). The heterogeneity analysis yielded an I^2^ value of 20.87% with a p-value of 0.00, indicating low to moderate heterogeneity among the studies.

**Fig 5:**
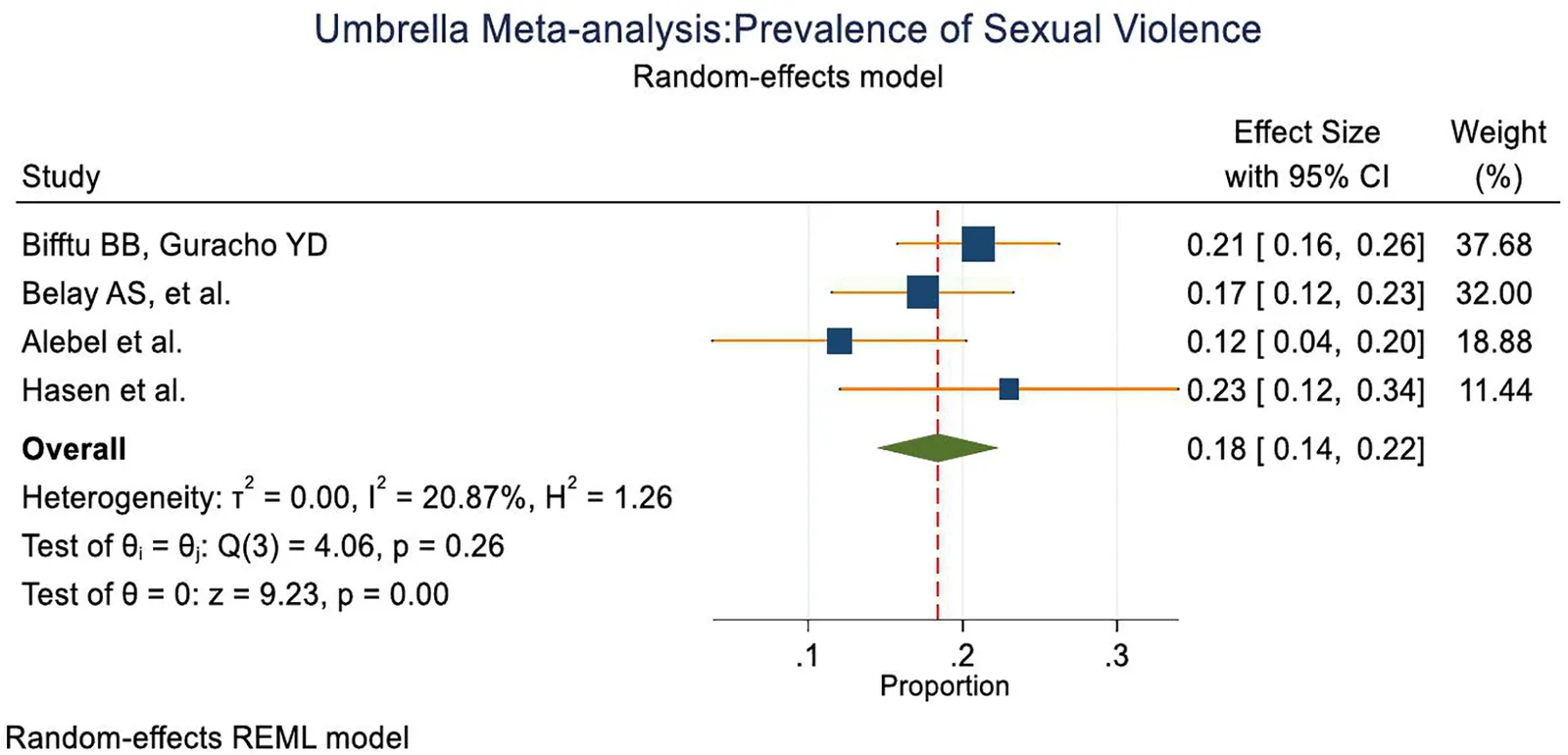
Pooled prevalence of partner sexual violence against women across included meta-analyses.

The pooled prevalence of partner physical violence in Ethiopia, based on four meta-analyses, was 19% (95% CI: 15–23) (Fig 6). The heterogeneity test indicated substantial variability among the included studies (I^2^ = 56.94%, p = 0.00). Given the presence of significant heterogeneity, a random-effects model was employed to estimate the pooled prevalence. This approach accounts for both within-study and between-study variability, thereby providing a more conservative and generalizable summary estimate.

**Fig 6:**
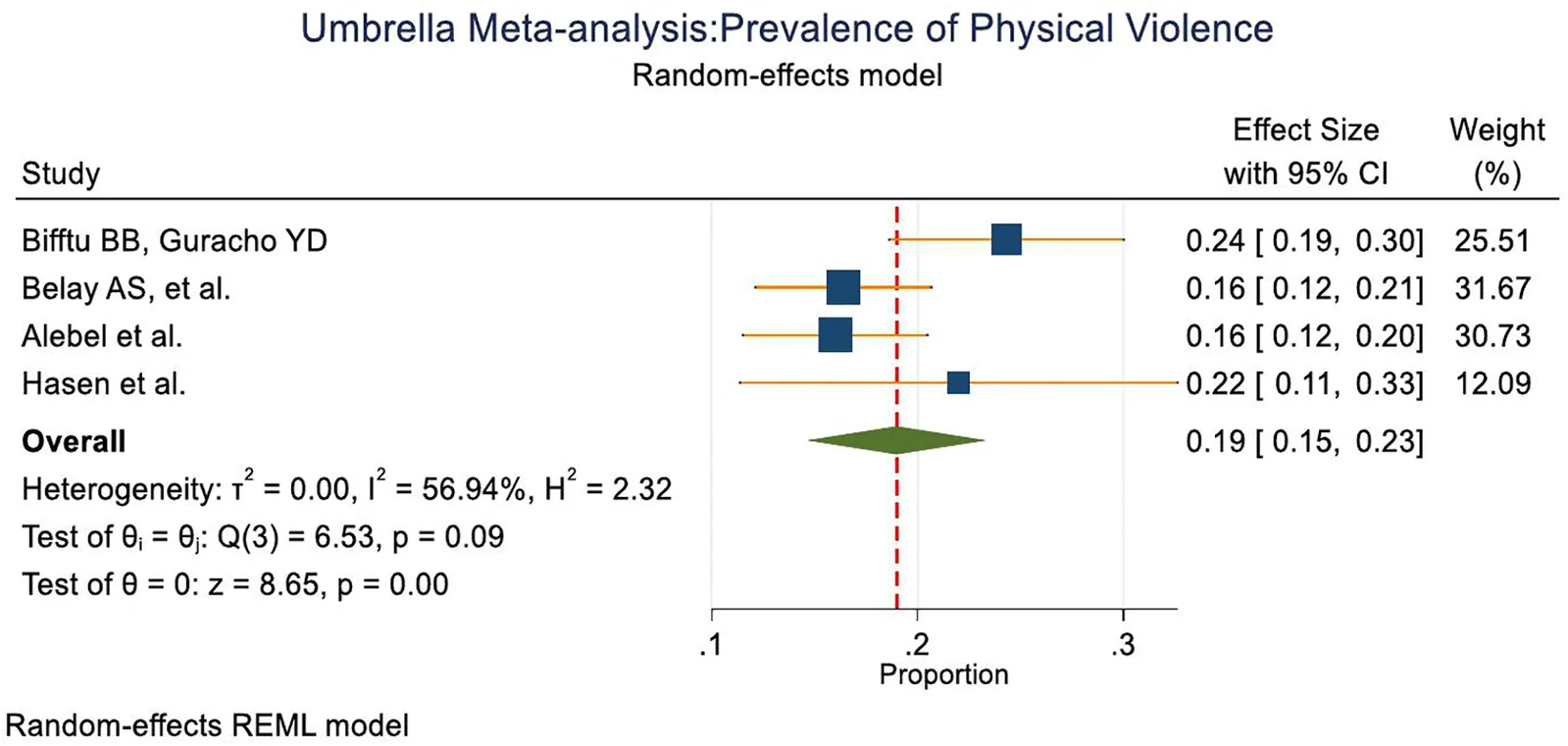
Umbrella review on the prevalence of partner physical violence.

Furthermore, the pooled prevalence of partner psychological violence in Ethiopia, based on four meta-analyses, was 27% (95% CI: 23–31) (Fig 7). The heterogeneity test indicated no variability among the included studies (I^2^ = 0.00%, p = 0.00).

**Fig 7:**
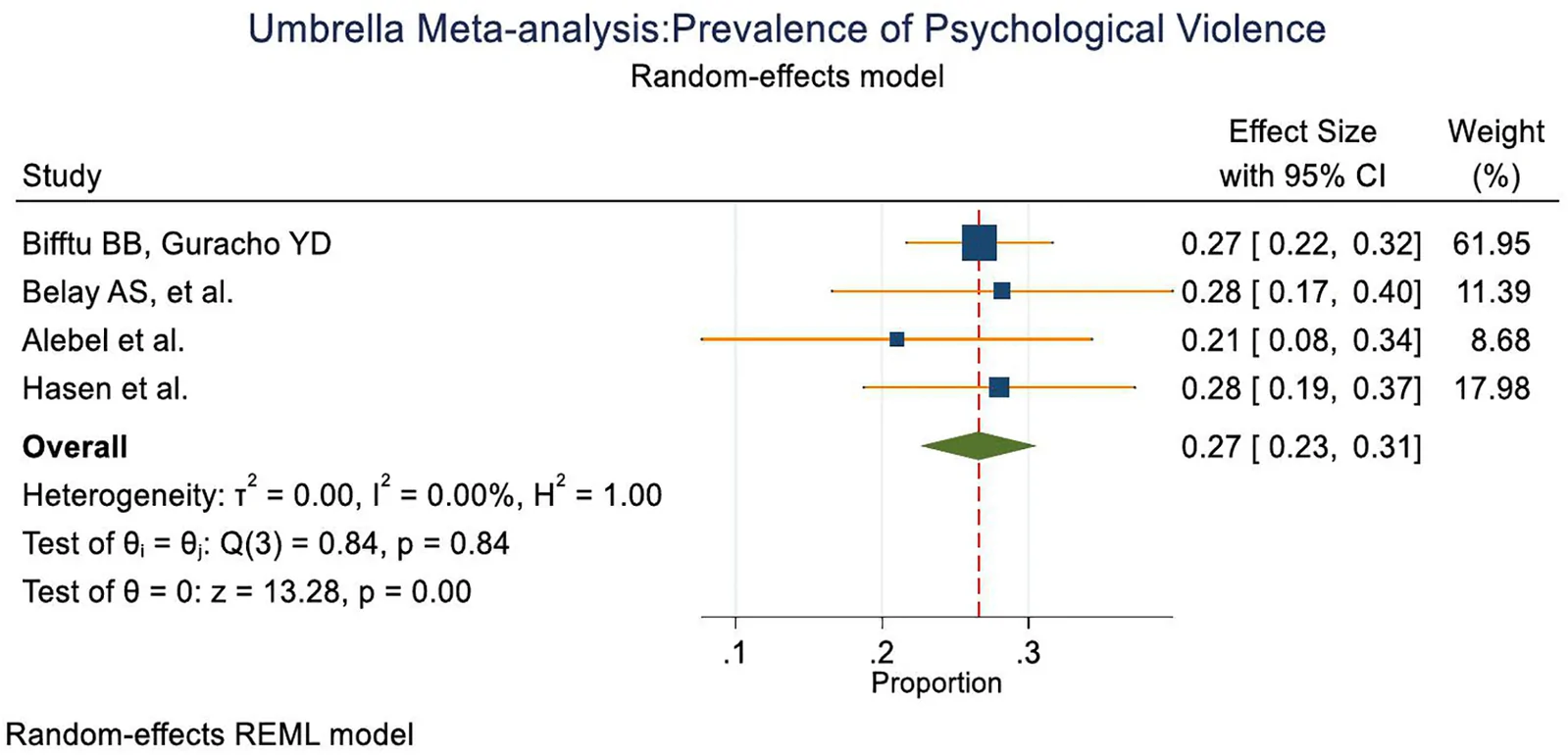
Pooled prevalence of partner psychological violence against women across included meta-analyses.

### Risk factors of intimate partner violence

The pooled adjusted odds ratio, based on a summary of six meta-analyses, indicated that women whose partners had lower educational status were more than twice as likely to experience IPV compared to their counterparts with higher educational attainment (AOR = 2.13, 95% CI: 1.22–3.69) (Fig 8).

**Fig 8:**
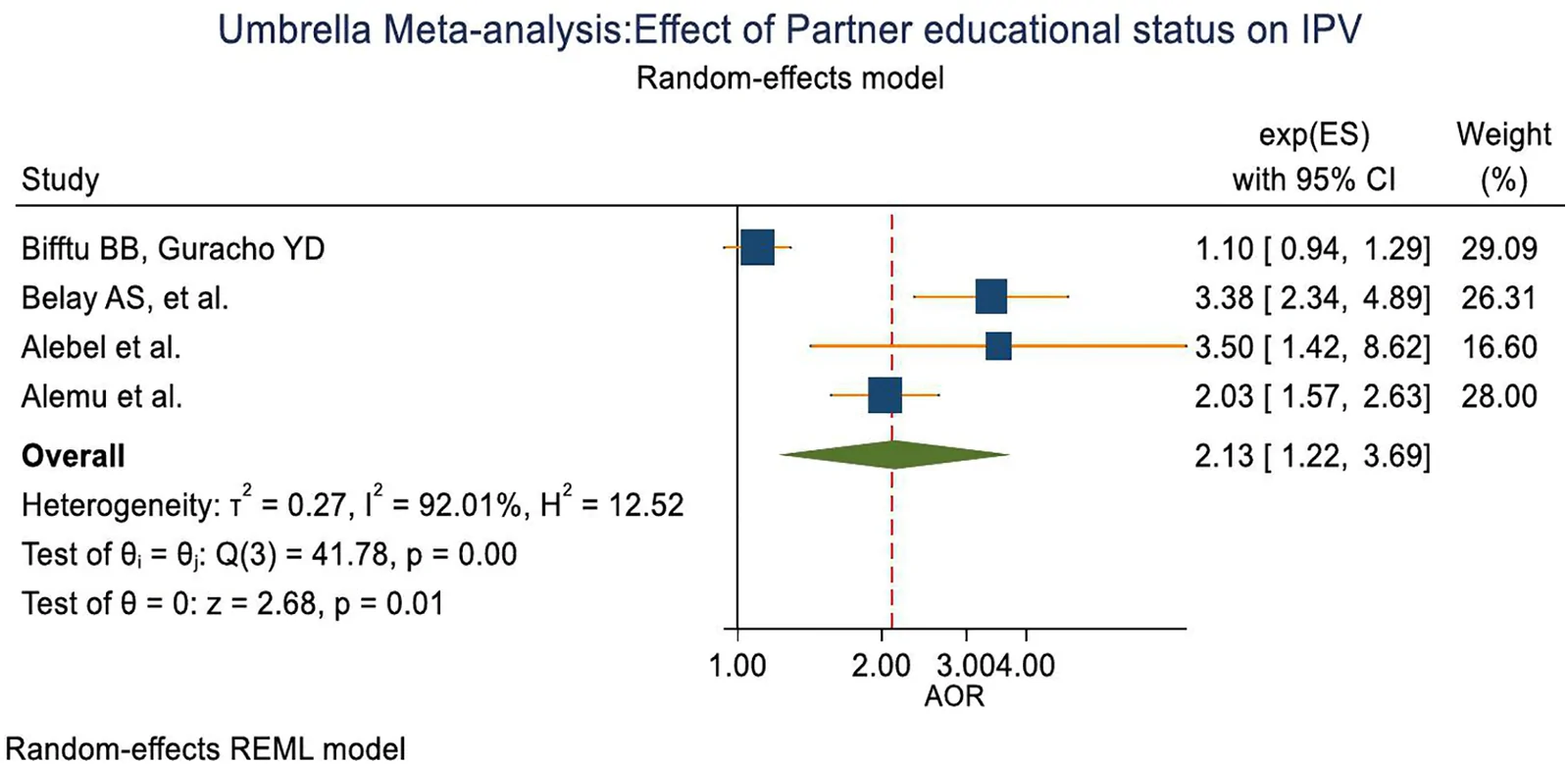
Umbrella review on the association between partner educational status and IPV.

The pooled AOR, based on a summary of three meta-analyses, indicated that women with lower educational status were more than twice as likely to experience IPV compared to their counterparts with higher educational attainment (AOR = 2.66, 95% CI: 1.99–3.56) (Fig 9).

**Fig 9:**
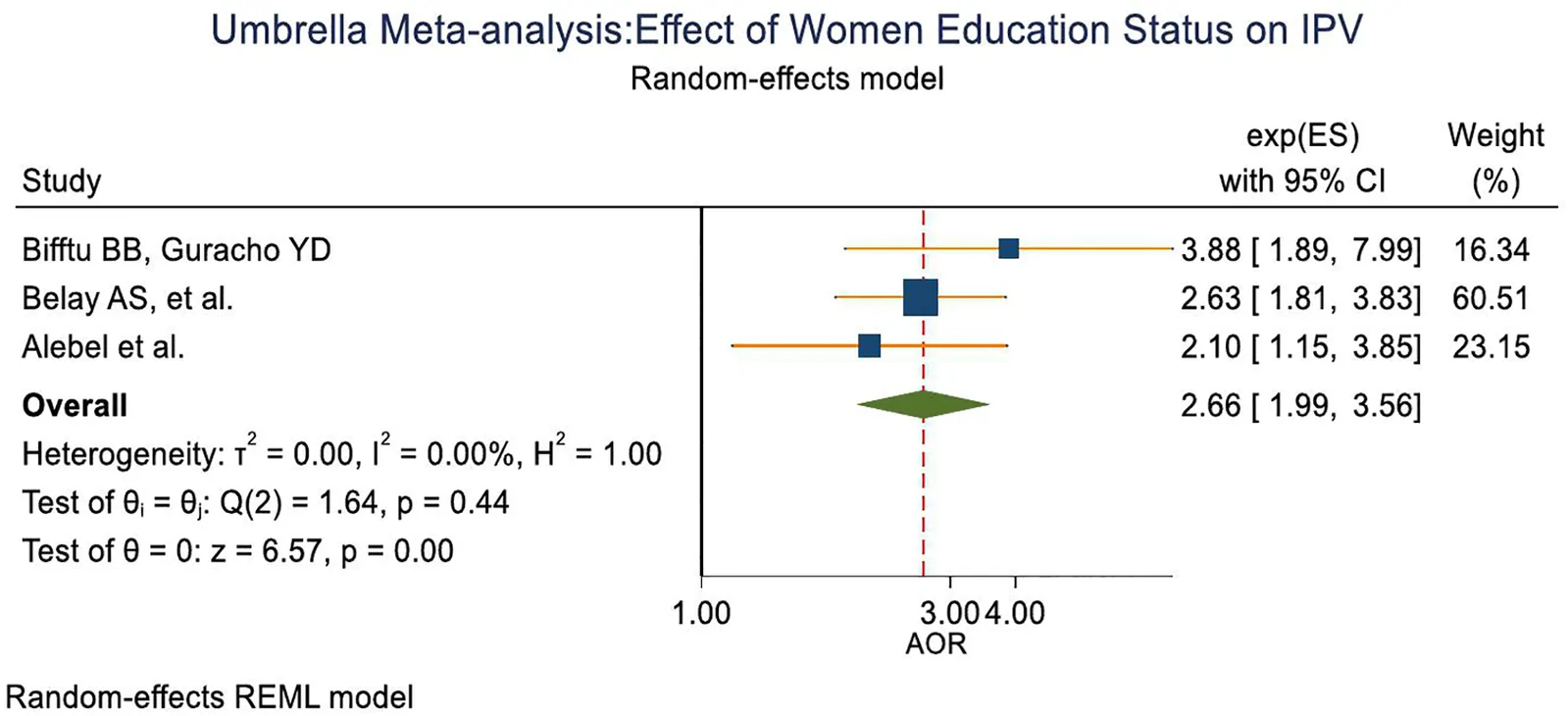
Umbrella review on the association between women education status and IPV.

The umbrella meta-analysis, based on five meta-analyses, showed that women whose partners consumed alcohol had more than twice the odds of experiencing IPV compared with those whose partners did not consume alcohol (AOR = 2.10, 95% CI: 1.83–2.40) (Fig 10). This finding indicates that partner alcohol consumption was significantly associated with an increased likelihood of IPV.

**Fig 10:**
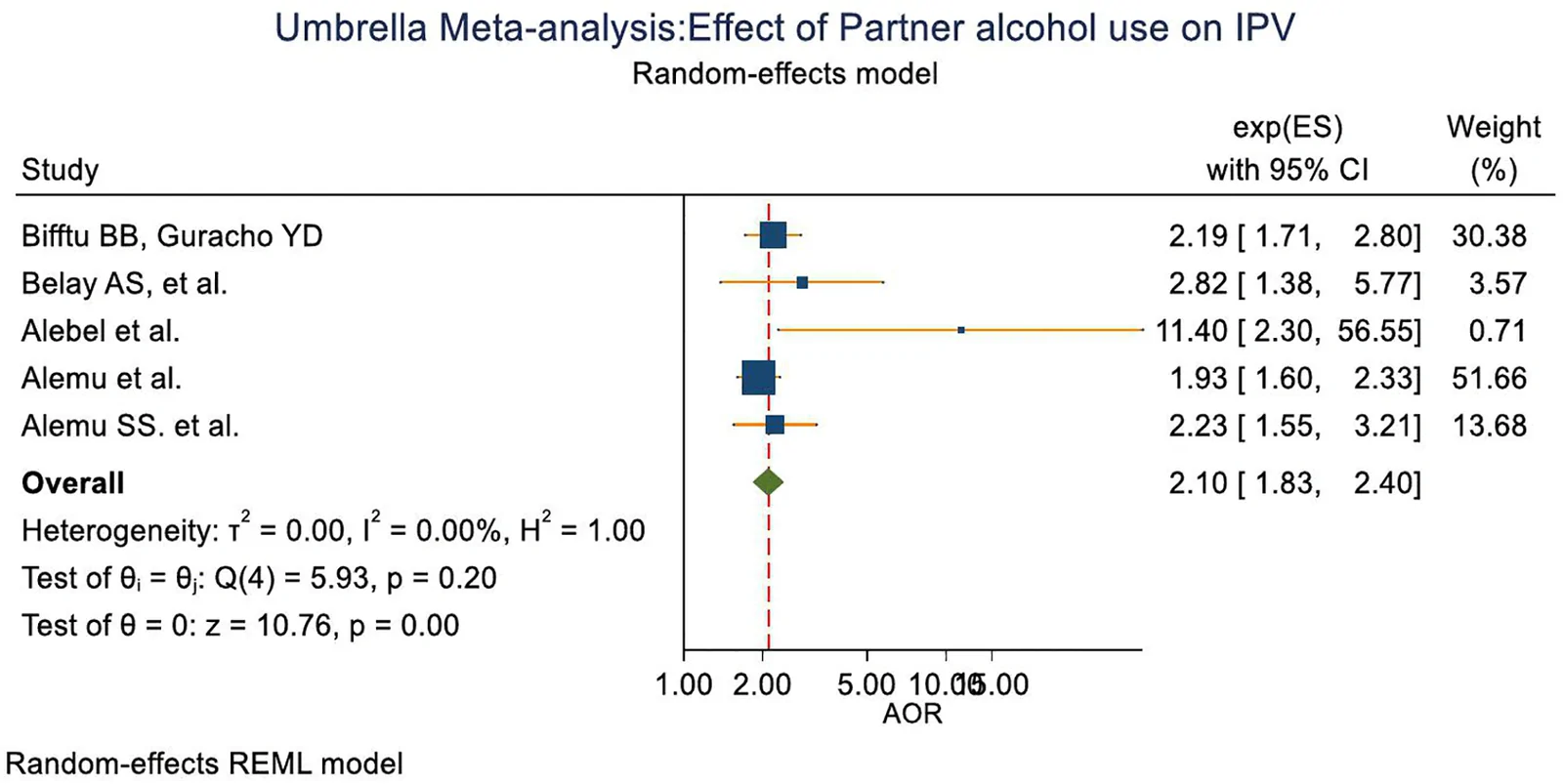
Umbrella review on the association between partner alcohol consumption and IPV.

Low decision-making power, defined as women's limited autonomy in making household decisions (e.g., decisions regarding healthcare, household purchases, or visits to family members), was also associated with IPV. The pooled analysis (Fig 11) showed that women with low decision-making power had 1.51 times higher odds of experiencing IPV than those with greater autonomy (AOR = 1.51, 95% CI: 1.09–2.09).

**Fig 11:**
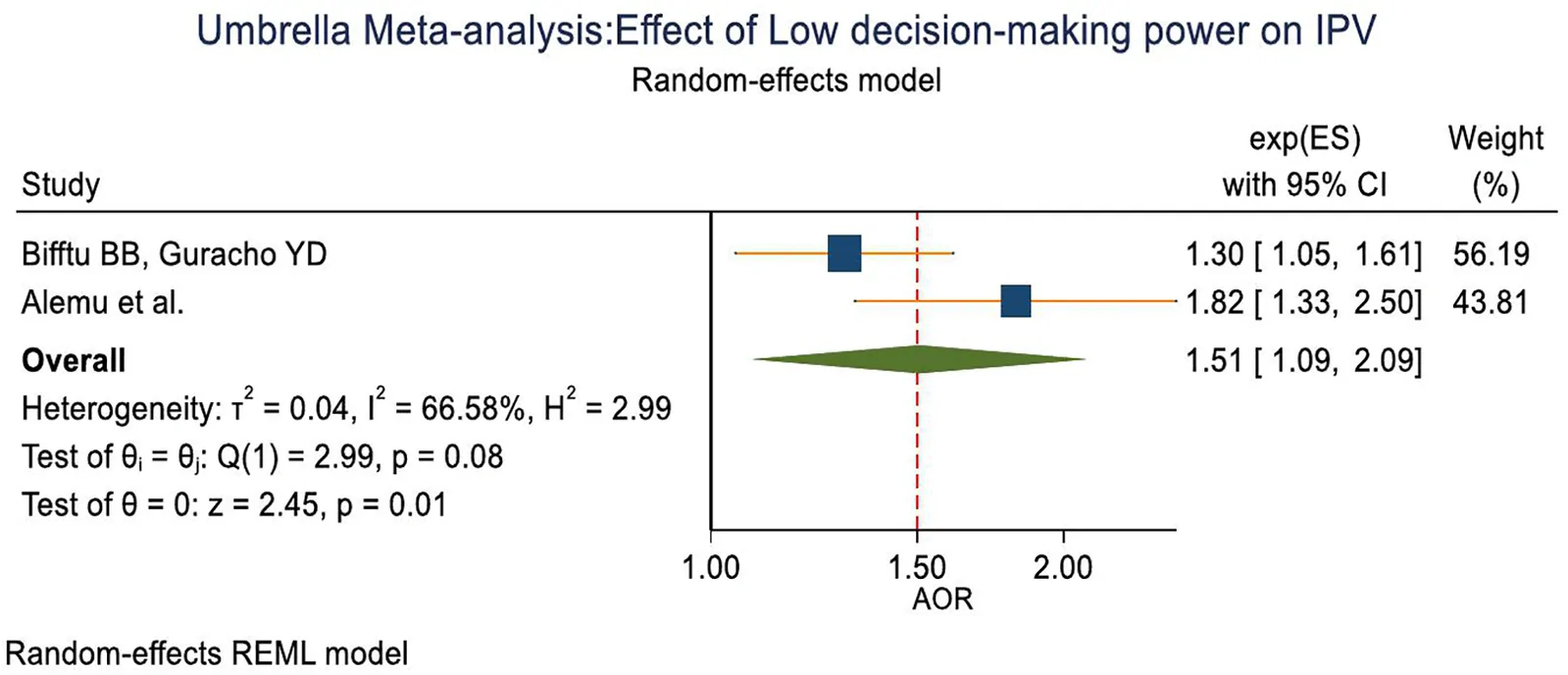
Umbrella review on the association between women decision making power and IPV.

The umbrella meta-analysis revealed that women whose partners used substances had 1.82 times higher odds of experiencing IPV compared to those whose partners did not use substances (AOR = 1.82, 95% CI: 1.55–2.14) (Fig 12).

**Fig 12:**
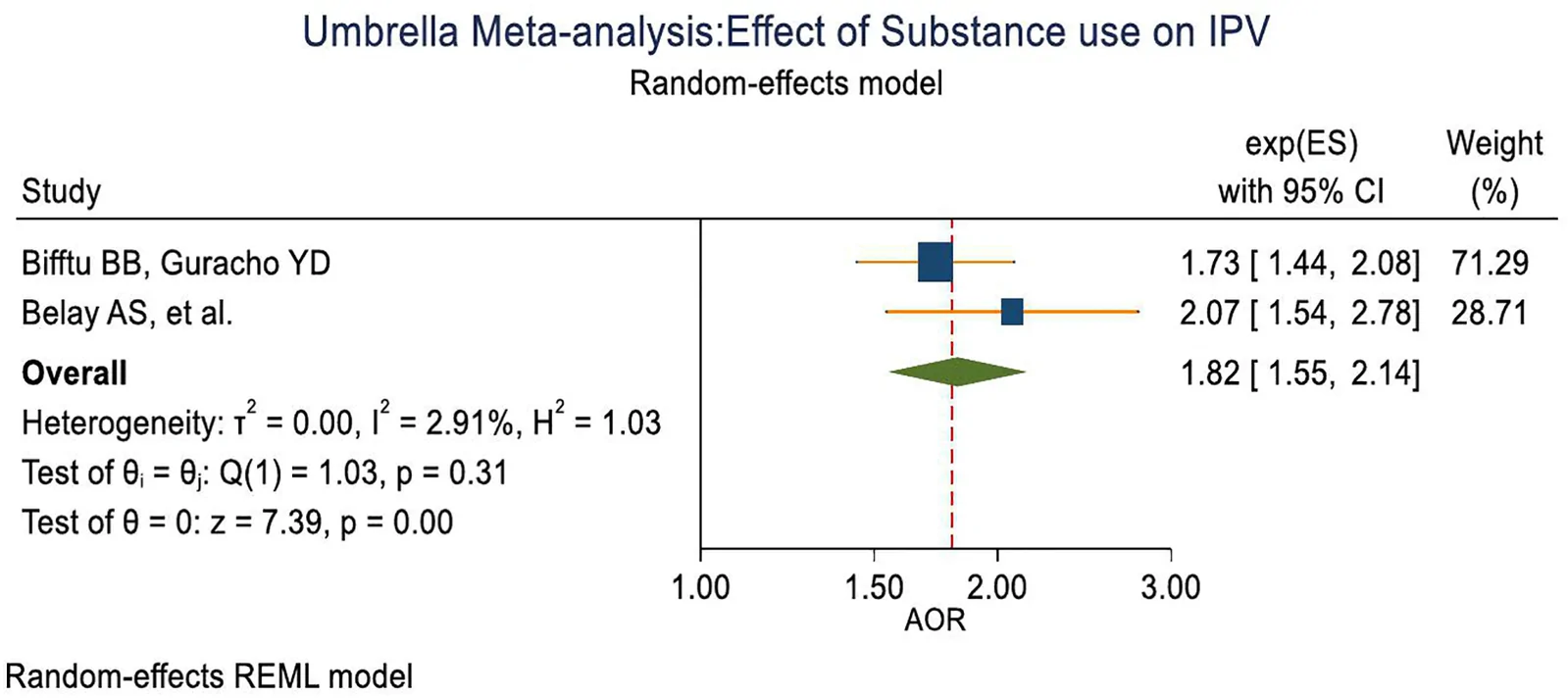
Umbrella review on the association between partner substance using and IPV.

The results presented in (Fig 13) showed that women who experienced income loss during the COVID-19 pandemic had higher odds of IPV than those whose income remained stable. The umbrella meta-analysis demonstrated a strong association between income loss and IPV (AOR = 9.86, 95% CI: 6.97–13.96).

**Fig 13:**
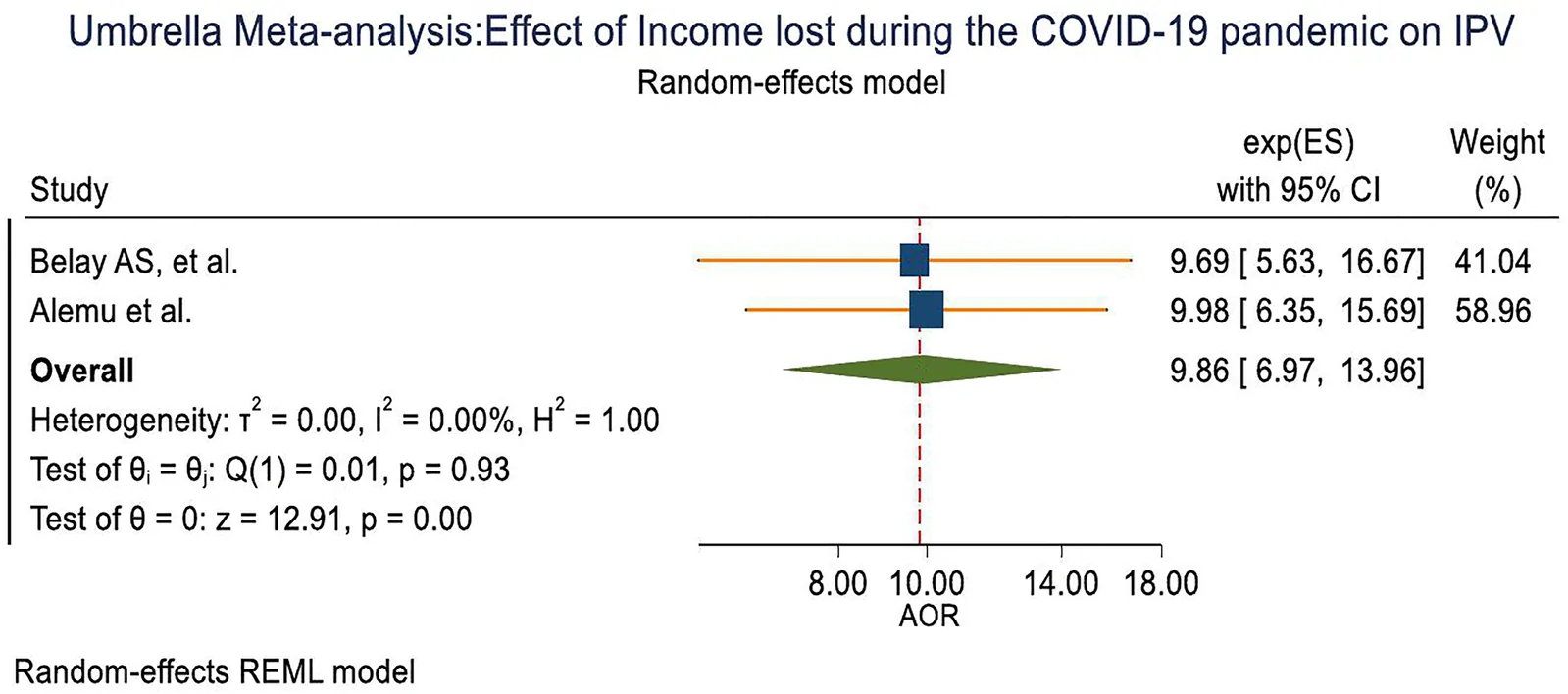
Umbrella review on the association between women income lost during the COVID-19 pandemic and IPV.

## Discussion

This umbrella review offers a comprehensive synthesis of evidence on the prevalence and predictors of IPV against women in Ethiopia, integrating findings from six systematic reviews and meta-analyses. Results show that IPV remains a substantial public health and human rights issue in Ethiopia, with a pooled prevalence of 35%. This estimate aligns with global evidence indicating that roughly one in three women worldwide experience IPV in her lifetime [1], and is comparable to estimates for sub-Saharan Africa, where IPV prevalence among women is approximately 36% [33]. These findings underscore the persistent, widespread nature of IPV in Ethiopia and the need for strengthened prevention and response strategies.

The continued high prevalence of IPV in Ethiopia, despite national legal frameworks and international commitments, suggests that existing prevention and response strategies have not been effectively implemented and should be interpreted within the country's sociocultural context. Weak enforcement of legislation, persistent patriarchal norms, unequal gender power relations, and women's economic dependence, combined with social acceptance of violence in some communities likely sustain this ongoing burden. Limited access to survivor-centered services, particularly in rural areas, along with the effects of conflict and displacement in some regions, may further heighten women's vulnerability to violence and reduce their opportunities to seek help.

The prevalence observed in this umbrella review falls within the broad range reported by previous studies in Ethiopia, which estimate that between 20% and 78% of women experience IPV during their lifetime [33]. This wide variation likely reflects differences in study settings, population characteristics, operational definitions of IPV, recall periods, measurement instruments, and cultural context.

Underreporting remains a major concern in IPV research, particularly in settings where stigma, fear of retaliation, economic dependence on partners, and limited support services discourage disclosure [34]. Evidence from the Ethiopian Demographic and Health Survey indicates that more than two-thirds of women who experience IPV do not seek help or disclose the violence a pattern likely driven by social acceptance of violence in some communities, and one that may further obscure the true magnitude of the problem [20].

This review also identified significant levels of IPV among specific subgroups of women. The pooled prevalence of IPV among pregnant women was 32%, indicating that violence during pregnancy remains a serious concern. Similar patterns have been observed elsewhere; systematic reviews from South Asia and Latin America report IPV prevalence among pregnant women ranging from 15% to 40%, suggesting that violence during pregnancy is a widespread global issue [35,36]. It poses serious risks to both maternal and neonatal health, including miscarriage, preterm birth, low birth weight, and adverse mental health outcomes [7,37].

Pregnancy represents a critical period during which women have frequent contact with healthcare providers, offering a valuable opportunity for routine IPV screening and early intervention. Failure to identify IPV during antenatal care may worsen both maternal and neonatal outcomes, underscoring the case for integrating IPV screening and response mechanisms into maternal and reproductive health services as a key prevention strategy [8,29].

This review also found a substantial prevalence of IPV during the COVID-19 pandemic, with an estimated pooled prevalence of 33%. This finding is consistent with global evidence describing the pandemic as a “shadow pandemic” of domestic violence [38]. A recent global meta-analysis reported that nearly 31% of women experienced IPV during the COVID-19 pandemic, with higher prevalence observed in developing regions compared with high-income countries. Pandemic-related restrictions, economic instability, and prolonged confinement within households have been identified as factors that may exacerbate existing vulnerabilities to violence against women [10,39,40]. These findings highlight the importance of maintaining continuity of support services, protection mechanisms, and IPV prevention and response programs for women during public health emergencies.

Analysis of different forms of IPV showed that psychological violence was the most common, with a prevalence of 27%, followed by physical violence (19%) and sexual violence (18%). These findings align with global literature indicating that psychological abuse is often the most prevalent yet underrecognized form of IPV [20]. Because it typically leaves no physical evidence, psychological violence including controlling behaviors, intimidation, and emotional abuse often remains invisible within health systems, despite its profound and long-lasting effects on women's mental health. Survivors frequently experience depression, anxiety, post-traumatic stress disorder, and suicidal behavior [3,6], underscoring the need for healthcare providers to be trained to recognize emotional abuse alongside physical and sexual violence.

This umbrella review also identified several important predictors of IPV in Ethiopia, reflecting the multifactorial nature of the problem. Educational status emerged as a significant predictor, with women who had lower levels of education being more than twice as likely to experience IPV compared with those who had higher education. Similarly, women whose partners had lower educational attainment also had increased risk of experiencing violence. These findings are consistent with previous research indicating that education plays a protective role by increasing awareness of rights, improving communication skills, and promoting gender equality within relationships [33].

Partner alcohol consumption was another strong risk factor identified in this review, with women whose partners consumed alcohol having more than twice the odds of experiencing IPV. This finding is widely supported by global literature linking alcohol misuse with aggressive behavior and increased likelihood of domestic violence [3,8,18]. Studies conducted in countries such as South Africa, India, and Brazil have consistently identified alcohol use as one of the strongest predictors of IPV [41]. Alcohol consumption may impair judgment, reduce impulse control, and escalate conflicts within intimate relationships, thereby increasing the likelihood of violent behavior [42].

Women’s autonomy within the household also emerged as a critical factor. Women with lower decision-making power had significantly higher odds of experiencing IPV compared with those who had greater autonomy. This finding reflects the influence of gender power imbalances and patriarchal social norms that restrict women's participation in household decision-making processes. In many societies, including Ethiopia, traditional gender roles may legitimize male dominance and contribute to the normalization of violence against women [20]. Addressing these structural inequalities is therefore essential for reducing IPV.

Several additional predictors were identified at the individual and relational levels, including childhood exposure to violence, family history of violence, supportive attitudes toward domestic violence, rural residence, and reproductive health factors such as unplanned pregnancy and delayed antenatal care initiation [27,28,32].

Collectively, these findings demonstrate that IPV is not driven by a single predictor but results from the interaction of individual, relational, community, and societal factors, consistent with the WHO ecological model of violence. This underscores that interventions focusing solely on individual behavior are unlikely to substantially reduce IPV without also addressing broader structural inequalities, and highlights the need for a comprehensive, multisectoral prevention approach.

The findings of this umbrella review have important implications for policy and practice in Ethiopia. Despite national and international initiatives addressing gender-based violence, including the Sustainable Development Goal 5 (SDG-5) agenda and the WHO RESPECT women framework implementation gaps remain significant [33,43]. Strengthening the health system's capacity to detect and respond to IPV is a critical first step: routine screening should be integrated into antenatal, reproductive health, and primary healthcare services, given that failure to identify IPV at these points of contact can worsen outcomes for both women and their children.

Beyond the health sector, community-based interventions aimed at transforming harmful gender norms, strengthening women's economic empowerment and education, and reducing harmful alcohol use among men should complement clinical responses. Multisectoral collaboration among the health, justice, education, and social protection sectors is essential for sustainable, long-term IPV prevention.

### Strengths and limitations of the study

A major strength of this review is that it is the first umbrella review to comprehensively synthesize evidence on the prevalence and predictors of IPV among women in Ethiopia. By integrating findings from multiple systematic reviews and meta-analyses, the review provides a higher level of evidence for policymakers and clinicians. Furthermore, methodological quality was assessed using AMSTAR-2, and overlap among primary studies was evaluated using a citation matrix and the CCA, enhancing the transparency of the synthesis.

Nevertheless, several limitations should be acknowledged. First, only six systematic reviews and meta-analyses were included, which may limit the breadth of the available evidence. Second, substantial heterogeneity was observed among the included reviews, likely reflecting differences in study populations, settings, outcome definitions, and measurement approaches. Third, a high degree of overlap among primary studies was identified (CCA = 12%), indicating that some studies contributed to more than one review, which may have resulted in partial double counting of evidence. Although this overlap was formally assessed and considered when interpreting the pooled estimates, it may still have influenced the results. Fourth, because fewer than ten reviews were included, a formal statistical assessment of publication bias was not performed.

Finally, a formal certainty-of-evidence assessment, such as GRADE, was not performed. Although the methodological quality of the included reviews was assessed using AMSTAR-2, the absence of a formal certainty assessment limits our ability to determine the overall confidence in the pooled estimates. Therefore, the findings should be interpreted cautiously, given the methodological limitations and heterogeneity of the available evidence.

## Conclusion

This umbrella review indicates that IPV remains a major public health and human rights concern in Ethiopia, affecting more than one-third of women. IPV occurs across various contexts, including during pregnancy and periods of socioeconomic crisis such as the COVID-19 pandemic, with psychological violence being the most commonly reported form, followed by physical and sexual violence. The review identified several factors consistently associated with IPV, including lower educational attainment, partner alcohol consumption, substance use, limited decision-making power, and economic instability.

These findings support the need for comprehensive, multisectoral strategies that address both structural and behavioral factors associated with IPV. Efforts to promote women’s education and empowerment, reduce harmful substance use, strengthen economic support programs, and integrate IPV screening and response into maternal and reproductive healthcare may contribute to reducing the burden of IPV. Where screening is implemented, it should be delivered by trained staff within a functioning, trauma-informed, survivor-centered response system that ensures confidentiality and privacy, includes immediate risk assessment and secure documentation, and provides informed choice, appropriate referral pathways, and access to survivor-centered support services. Strengthening the implementation of existing policies and expanding survivor-centered services could further support progress toward Sustainable Development Goal 5, which aims to eliminate all forms of violence against women and girls.

However, these findings should be interpreted with caution because only six systematic reviews and meta-analyses were included, substantial heterogeneity was observed, and a high degree of overlap among primary studies was identified (CCA = 12%), which may have resulted in partial double counting of evidence. In addition, although the methodological quality of the included reviews was assessed using AMSTAR-2, a formal certainty-of-evidence assessment, such as GRADE, was not performed. These limitations should be considered when interpreting the overall strength and reliability of the evidence.

## Supporting information

S1 Table PRISMA 2020 Checklist followed for this systematic review and meta-analysis. (DOCX)

S2 Table Citation matrix of primary study overlaps among included systematic reviews and meta-analyses. (DOCX)

S3 Table Methodological quality assessment of included systematic reviews and meta-analyses using AMSTAR-2. (DOCX)

## Abbreviations

AMSTAR-2: A Measurement Tool to Assess systematic Reviews
AOR: Adjusted Odds Ratio
CCA: Corrected Covered Area
IPV: Intimate Partner Violence
SRMAs: Systematic Reviews and Meta-Analyses
