# Supplementary material for: Intimate partner violence against women in Ethiopia: An umbrella review of systematic reviews and meta-analyses
Source: PLoS One. 2026 Nov 11;21(11):e0362748. doi: 10.1371/journal.pone.0362748 (PMC13754129; doi:10.1371/journal.pone.0362748)
Supplement: Supplementary file 2 [file pone.0362748.s002.docx]

**S2 Table. Citation matrix of primary study overlaps among included systematic reviews and meta-analyses.**

| **S/No** | **Primary study (Author, Year)** | **SRMA 1** | **SRMA 2** | **SRMA 3** | **SRMA 4** | **SRMA 5** | **SRMA 6** |
| --- | --- | --- | --- | --- | --- | --- | --- |
| 1. | Ashenafi W (2020) | ✓ |  |  |  | ✓ |  |
| 2. | Belay LM (2018) | ✓ |  |  |  |  |  |
| 3. | Gashaw (2019) | ✓ |  |  |  |  |  |
| 4. | Belay S (2019) | ✓ |  |  |  | ✓ |  |
| 5. | Yehunnes (2019) | ✓ |  |  |  |  |  |
| 6. | Azene (2019) | ✓ |  |  |  | ✓ |  |
| 7. | Berhanie (2019) | ✓ |  |  |  | ✓ |  |
| 8. | Gebreselasie (2020) | ✓ |  |  |  |  |  |
| 9. | Lencha (2019) | ✓ |  |  |  |  |  |
| 10. | Abdurashid (2013) | ✓ |  | ✓ |  |  |  |
| 11. | Tulu (2019) | ✓ |  |  |  |  |  |
| 12. | Adhena (2020) | ✓ |  |  |  | ✓ |  |
| 13. | Tadesse (2020) | ✓ |  |  |  | ✓ |  |
| 14. | Eba (2014) | ✓ |  |  |  |  |  |
| 15. | Laelago (2017) | ✓ |  |  |  | ✓ |  |
| 16. | Mussa (2020) | ✓ |  |  |  |  |  |
| 17. | Abate (2016) | ✓ |  | ✓ |  | ✓ |  |
| 18. | Yimer (2014) | ✓ |  | ✓ |  | ✓ |  |
| 19. | Gebrezgi (2017) | ✓ |  | ✓ |  | ✓ |  |
| 20. | Fekadu (2018) | ✓ |  |  |  |  |  |
| 21. | Mohammed (2017) | ✓ |  |  |  |  |  |
| 22. | Demelash (2015) | ✓ |  | ✓ |  | ✓ |  |
| 23. | Bifftu (2017) | ✓ |  | ✓ |  | ✓ |  |
| 24. | Kassa (2016) | ✓ |  | ✓ |  |  |  |
| 25. | Gossaye (2003) | ✓ |  |  |  |  |  |
| 26. | Nigussie (2020) | ✓ |  |  |  |  |  |
| 27. | Belay AS (2022) |  | ✓ |  | ✓ |  |  |
| 28. | Tadesse AW (2022) |  | ✓ |  | ✓ |  |  |
| 29. | Engeda AS (2022) |  | ✓ |  | ✓ |  |  |
| 30. | Kassie A (2022) |  | ✓ |  | ✓ |  |  |
| 31. | Gebrewahd GT (2020) |  | ✓ |  | ✓ |  |  |
| 32. | Fetene G (2022) |  | ✓ |  | ✓ | ✓ |  |
| 33. | Demeke MG (2023) |  | ✓ |  | ✓ |  |  |
| 34. | Asratie MH (2022) |  | ✓ |  |  |  |  |
| 35. | Wood SN (2022) |  | ✓ |  | ✓ |  |  |
| 36. | Shitu S (2021) |  | ✓ |  | ✓ |  |  |
| 37. | Teshome A (2021) |  | ✓ |  | ✓ |  |  |
| 38. | Mingude AB (2021) |  | ✓ |  |  |  |  |
| 39. | Laelago (2014) |  |  | ✓ |  |  |  |
| 40. | Musa (2021) |  |  |  |  | ✓ |  |
| 41. | Deyessa (2016) |  |  |  |  | ✓ |  |
| 42. | Atsbaha (2022) |  |  |  |  | ✓ |  |
| 43. | Gashaw (2018) |  |  |  |  | ✓ |  |
| 44. | Goyomsa (2022) |  |  |  |  |  | ✓ |
| 45. | Diribe (2016) |  |  |  |  |  | ✓ |
| 46. | Alemie (2023) |  |  |  |  |  | ✓ |
| 47. | Biranu (2023) |  |  |  |  |  | ✓ |
| 48. | Meskele (2019) |  |  |  |  |  | ✓ |
| 49. | Beletu (2022) |  |  |  |  |  | ✓ |
| 50. | Deyessa (2018) |  |  |  |  |  | ✓ |

✓ Indicates that the primary study was included in the corresponding systematic review/meta-analysis.

**Note:** The Corrected Covered Area (CCA) was calculated based on 50 unique primary studies and 80 study occurrences across the six included systematic reviews and meta-analyses. The resulting CCA was 12%, indicating a high degree of overlap.

- Number of included reviews (c) = 6
- Number of unique primary studies (r) = 50
- Total number of study occurrences across all reviews (N) = 80 (i.e., the total number of check marks in the citation matrix)

The CCA was calculated using the following formula:

CCA = (N − r) / (rc − r) = (80 − 50) / (50 × 6 − 50) = 12%
