# Supplementary material for: Intimate partner violence against women in Ethiopia: An umbrella review of systematic reviews and meta-analyses
Source: PLoS One. 2026 Nov 11;21(11):e0362748. doi: 10.1371/journal.pone.0362748 (PMC13754129; doi:10.1371/journal.pone.0362748)
Supplement: Supplementary file 3 [file pone.0362748.s003.docx]

**S3 Table. Methodological quality assessment of included systematic reviews and meta-analyses using AMSTAR-2.**

| **Authors** | **AMSTAR Items*** | | | | | | | | | | | | | | | | | |  |
| --- | --- | --- | --- | --- | --- | --- | --- | --- | --- | --- | --- | --- | --- | --- | --- | --- | --- | --- | --- |
|  | **Year** | **1** | **2** | **3** | **4** | **5** | **6** | **7** | **8** | **9** | **10** | **11** | **12** | **13** | **14** | **15** | **16** | **Total** | **Overall AMSTAR-2 rating** |
| Bifftu BB, Guracho YD | 2022 | 1 | 0 | 2 | 2 | 2 | 2 | 0 | 1 | 1 | 0 | 2 | 1 | 2 | 2 | 2 | 2 | 22 | Low |
| Belay AS, et al. | 2025 | 2 | 1 | 2 | 2 | 2 | 2 | 0 | 2 | 1 | 2 | 2 | 2 | 2 | 2 | 2 | 2 | 28 | Moderate |
| Alebel et al. | 2018 | 2 | 1 | 2 | 2 | 2 | 1 | 0 | 2 | 2 | 2 | 1 | 2 | 2 | 2 | 2 | 2 | 27 | Moderate |
| Alemu et al. | 2024 | 1 | 1 | 2 | 2 | 2 | 2 | 0 | 2 | 2 | 2 | 2 | 2 | 2 | 2 | 2 | 2 | 27 | Moderate |
| Belay et al. | 2022 | 1 | 0 | 2 | 2 | 2 | 2 | 0 | 1 | 2 | 2 | 2 | 2 | 2 | 2 | 2 | 2 | 26 | Low |
| Alemu SS. et al. | 2025 | 2 | 2 | 2 | 2 | 2 | 2 | 0 | 2 | 2 | 2 | 2 | 2 | 2 | 2 | 2 | 2 | 30 | High |

*****Individual items on the AMSTAR-2 checklist were scored on a scale from 0 to 2. A score of 1 indicated partial adherence to methods that reduce bias, while a score of 2 was assigned for the full application of such methods.

For a comprehensive description of each assessment item, the official AMSTAR-2 checklist is available online (<https://amstar.ca/Amstar_Checklist.php>).
